# Muscle fatigue and muscle weakness: what we know and what we wish we did

**DOI:** 10.3389/fphys.2013.00125

**Published:** 2013-05-30

**Authors:** Christina Karatzaferi, P. Bryant Chase

**Affiliations:** ^1^Department of Physical Education and Sports Science, University of ThessalyTrikala, Greece; ^2^Department of Kinesiology, Center for Research and TechnologyThessaly, Trikala, Greece; ^3^Department of Biological Science, The Florida State UniversityTallahassee, FL, USA

This Research Topic on muscle fatigue and muscle weakness presents the latest ideas, arguments, and evidence from investigations at the molecular level to macroscopic observations on whole animals including humans, in an effort to identify critical factors underlying fatigue and weakness in health and disease. Skeletal muscles confer movement to the human body using vast amounts of energy provided through complex metabolic pathways such that whole body mobility and energy balance are largely dictated by muscle activity. Conversely, muscle function reflects overall health status as exercise history and chronic conditions affect either or both muscle quality, including protein and fat content, and muscle mass. In health, muscle fatigue is temporary and recovery occurs rapidly, and recreational or competitive athletes are always pursuing the next best fatigue “fix.” However, after inactivity—whether due to lifestyle choices, injury or chronic disease—muscle fatigue may occur prematurely and persist, endangering a person's safety because weakness can lead to falls that may result in loss of independence. Individuals are then trapped in a self-perpetuating, vicious cycle of inactivity, disuse muscle atrophy/weakness, and metabolic disturbance that compounds morbidity (i.e., causing metabolic syndrome, obesity, hypertension, cachexia) and eventually premature death. Such issues transcend many scientific disciplines and it becomes evident that not only recognizing fundamental factors in muscle fatigue and muscle weakness is necessary, but also evaluating their interaction with factors outside of the muscle is essential if we aspire to design better interventions that improve muscle function and thus improve quality of life and life prognosis for the ageing population and chronic disease patients.

Fatigue and weakness may stem from changes within myocytes that affect cross-bridge function or Ca^2+^ activation, to changes within the circulation or function of the nervous system. Within myocytes, metabolic products of ATP hydrolysis in the cytoplasm such as inorganic phosphate (Pi), protons (H^+^ or pH), and ADP have often been considered as agents that could disrupt force generation at the sarcomere level (Fabiato and Fabiato, 1978; Cooke and Pate, 1985; Metzger and Moss, 1987; Nosek et al., 1987, 1990; Chase and Kushmerick, 1988, 1995; Cooke et al., 1988; Godt and Nosek, 1989; Pate and Cooke, 1989; Metzger and Moss, 1990a,b; Pate et al., 1995, 1998; Wiseman et al., 1996; Karatzaferi et al., 2003, 2008). These effects may be due to direct binding to proteins, or due to a more global alteration of cellular energetics (ΔG_ATP_) in the myocyte (Karatzaferi et al., 2004).

In this Research Topic, Debold (2012) consolidates the most recent information, including single molecule assays and molecular biological approaches, about the mechanisms by which Pi, H^+^, and ADP inhibit actomyosin cross-bridge cycling and thin filament Ca^2+^-activation. Allen and Trajanovska (2012) provide a synthesis on the multiple roles of Pi in fatigue, including novel results from their group, showing that Pi is even more detrimental when its effects on Ca^2+^ release are combined with inhibition of actomyosin force generation and Ca^2+^ activation. In addition to activity-driven changes in metabolites and cellular energetics, mutations in sarcomeric proteins have been associated with prolonged muscle weakness in myopathies. Moving away from actomyosin events, Ottenheijm et al. (2012) consider the role of nebulin in sarcomere function, and how transgenic mouse models can inform us about mutations in the giant filamentous protein nebulin, and mutations in other thin filament and closely related proteins that are associated with nemaline myopathy.

To fully test our understanding of muscle fatigue, appropriately detailed models of muscle function will be necessary. Röhrle et al. (2012) make major advances in that arena by presenting a multi-scale, finite element model of the human tibialis anterior. Their model has the advantage of allowing simulation of fatigue at the cellular and motor unit levels, and can incorporate altered recruitment patterns of motor units due to central components of fatigue. Thus their model can serve an invaluable role as we bridge our understanding between the cellular and tissue levels.

Muscle's plasticity is most readily evident in its adaptation to repeated exercise, and conversely to inactivity that may be associated with various injuries and disease states. Bogdanis (2012) reviews the long-term changes in muscle at the molecular, cellular, and tissue levels, as well as the corresponding functional changes that are associated with these adaptations to activity level history. Fatigability is a key functional characteristic of different muscle fiber types, and can vary greatly with activity, or inactivity, and Bogdanis evaluates the utility of high-intensity bouts of exercise for modulating fatigability by training, or as a component in therapy. Bogdanis' section on effects of reactive oxygen species (ROS) sets the stage for the succinct review on antioxidants by Hernández et al. (2012). Despite the popularity of antioxidants as nutritional supplements, Hernández et al. report that their utility for either minimizing or speeding recovery from fatigue appears to be limited to specific muscle types. Moreover, Bogdanis' (2012) section on neural factors opens the discussion on the role of non-muscle factors in fatigue and serves as a bridge to the articles by Kobilo and van Praag (2012), Sakkas and Karatzaferi (2012), and Noakes (2012).

What is the extent to which muscle activity and fatigue influence the function of other physiological systems of the body, particularly the nervous system upon which skeletal muscle depends for activation, and how much of fatigability is determined centrally? In the commentary by Kobilo and van Praag (2012), pharmacological activation of AMP-activated protein kinase (AMPK)—a metabolic regulator that is activated during exercise—is shown to alter performance in a test of spatial memory and hippocampal neurogenesis in mice in a time-dependent manner. How can diseases and treatments modify the experience and presentation of fatigue? In their opinion article, Sakkas and Karatzaferi (2012) consider available evidence on the complex symptomatology of fatigue in renal patients on hemodialysis treatment. By drawing analogies to Chronic Fatigue Syndrome, Sakkas and Karatzaferi (2012) present the view that fatigue, as experienced by patients undergoing routine hemodialysis, might be better addressed by caregivers as a syndrome and not with isolated measures since its apparent complexity requires a cross-disciplinary therapeutic approach. While hemodialysis and some other patients may be afflicted with specific syndromes, the rest of us have all heard the expression “mind over matter.” Does it apply to muscle? Noakes (2012) concludes the series with a challenging review, partly historical in nature, arguing that the key component in fatigue is central. The author discusses the accepted models on the limits of human exercise performance, and presents his central governor model of exercise regulation, arguing that fatigue is brain-derived, being an important homeostatic mechanism that protects an organism from catastrophic overexertion.

It is our sincere hope that this Research Topic will not only provide readers with new insights and viewpoints on the issue of muscle fatigue and weakness, but will also stimulate novel ideas, experiments, and further advances in this research field.
